# *Helicobacter pylori* associated factors in the development of gastric cancer with special reference to the early-onset subtype

**DOI:** 10.18632/oncotarget.25757

**Published:** 2018-07-24

**Authors:** Małgorzata Pucułek, Julita Machlowska, Ryszard Wierzbicki, Jacek Baj, Ryszard Maciejewski, Robert Sitarz

**Affiliations:** ^1^ Department of Human Anatomy, Medical University of Lublin, Poland; ^2^ Department of Surgery with Trauma, Orthopaedic and Urological Subunit, Independent Public Health Care Center of the Ministry of Interior and Administration in Lublin, Poland; ^3^ Department of Surgical Oncology, Medical University of Lublin, Poland; ^4^ Department of Surgery, St. John's Cancer Center, Lublin, Poland

**Keywords:** early-onset gastric cancer, gastric cancer, H. pylori, genotypes, virulence factors

## Abstract

Nowadays, gastric cancer is one of the most common neoplasms and the fourth cause of cancer-related death on the world. Regarding the age at the diagnosis it is divided into early-onset gastric carcinoma (45 years or younger) and conventional gastric cancer (older than 45). Gastric carcinomas are rarely observed in young population and rely mostly on genetic factors, therefore provide the unique model to study genetic and environmental alternations. The latest research on early-onset gastric cancer are trying to explain molecular and genetic basis, because young patients are less exposed to environmental factors predisposing to cancer. In the general population, *Helicobacter pylori*, has been particularly associated with intestinal subtype of gastric cancers. The significant association of *Helicobacter pylori* infection in young patients with gastric cancers suggests that the *bacterium* has an etiologic role in both diffuse and intestinal subtypes of early-onset gastric cancers. In this paper we would like to ascertain the possible role of *Helicobacter pylori* infection in the development of gastric carcinoma in young patients. The review summarizes recent literature on early-onset gastric cancers with special reference to *Helicobacter pylori* infection.

## INTRODUCTION

Gastric cancer (GC) is a multifactorial disease in which both genetic and environmental factors are involved. According to the statistic, GC is the fourth cause of cancer death worldwide, with a median overall survival of ≤12 months for advanced stage [1]. It is rare in the younger population, where less than 10% of patients suffer from GC before 45 years of age [2–6]. It is highly heterogeneous disease with different molecular and genetic alterations [7].

The most common classification of gastric cancer is the Lauren classification. It differentiates two types of GC - intestinal and diffuse which have distinguishing features like morphology, genetics, clinical characteristics, progression pattern and epidemiology [8]. Diffuse-type GC is composed of poorly cohesive single cells without gland formation. Intestinal-type GC consists glandular or tubular components with various degrees of differentiation [9, 10]. While the incidence of GC has decreased worldwide, the incidence of GC with signet-ring cell is rising. In the past, GC with signet-ring cell was classified as “diffuse type” according to Lauren’s classification [8]. Now, signet-ring cell carcinoma is defined as a poorly cohesive carcinoma composed predominantly of tumor cells with prominent cytoplasmic mucin and a crescent-shaped nucleus eccentrically placed [11]. It is important to understand that not all gastric cancers classified as “undifferentiated” or “diffuse” are signet-ring cell cancers.

Gastric cancer is a multifactorial disease, whereas *Helicobacter pylori (H. pylori)* infection is one of the risk factors in developing the cancer, but not exclusive. The infection with *H. pylori* increases the risk of developing GC about six-fold [10, 12]. Therefore, the World Health Organization (WHO) classified *H. pylori* as a class I carcinogen in 1994 [10, 13]. *H. pylori*, also known as *Campylobacter pylori*, is a *bacterium* detected in 1982 by Australian scientists in human mucous membrane in digestive system [14]. The name of this pathogen, from Latin, in which helix means spiral, refers to the characteristic shape of it, which probably facilitates its penetration into the mucous membrane of the digestive system. The second part of the name of *H. pylori* comes from pylorus because the *bacteria* is detected primarily in the distal part of the stomach - the pylorus. It was found that the gastric acid does not damage *H. pylori* and it even seemed to be essential to its growth. Later, it was shown that *H. pylori* could also infect other tissues, e.g. the liver or the eye [15, 16].

In further studies, it was found that there was a relationship between the presence of *H. pylori* in the upper gastrointestinal tract and gastric and duodenal ulcers. However, that bacterial infection alone is not synonymous with the development of peptic ulcer disease. Only a small percentage of infected people will have gastric cancer. *H. pylori* infection has been shown to range from approximately 60% in the general population to approximately 84% in patients with gastric cancer [17]. There must be other critical cofactors affecting risk, which may also explain the difference in morbidity between races and sex. Possible factors are specific genetic alterations, age at the onset of infection by *H. pylori*, differences in gastric acidity, and environmental factors like diet including salt consumption and smoking [17]. Nearly half of humanity (more in developing countries) is infected with *H. pylori* (in Poland about 80% of adults and about 30% of children) [18]. Meanwhile, 5-10% of the adult population falls ill for peptic ulcer of the stomach and duodenum. This means that only about one in ten people infected with *H. pylori* will develop peptic ulcer disease. In addition, *H. pylori* is not found in some people suffering from peptic ulcer disease. Therefore, *H. pylori* cannot be the cause of all gastric and duodenal ulcers, and alone cannot explain the pathogenesis of gastric carcinoma [19].

The review summarizes the data about early-onset gastric cancer (EOGC) and the role of *H. pylori* in the developing this subtype of gastric carcinoma.

### Epidemiology of *H. pylori* infection in Europe and Asia regions

Various *H. pylori* infection prevalence was noted, based on the study region and period of such statistics. The infection ratio is based mostly on the hygienic lifestyle standards, which are better in Europe. Screening investigations are mostly performed in Asia regions, in Europe the infection control is decreased, as the disease rates are rather low, and the interventions are extravagant [20]. Better controlling of the lifestyle agents may cause a significant reduction of *H. pylori* associated diseases, like stomach cancer.

It was postulated as the lowest supremacy of infection was found in Northern Europe, the highest were in Southern and Eastern Europe [21]. The total prevalence of *H. pylori* infection in adults European is about 20%- 40% [22]. The studies on epidemiology among European population showed no important difference of *H. pylori* infection correlated with the gender. If there was a gender difference, the infection was increased in men. However, the age is affected with the *H. pylori* action, occurring mostly in older age groups [23]. There are some exceptions, like the Spanish investigation, which displayed lower infection ration among oldest individuals [24]. Additionally, in Swedish study, blood donors infected with the *bacterium* showed decreasing level after age of 50 [25]. In Southern and Eastern Europe, the infection of *H. pylori* is remarkably higher in comparison to other parts of the continent, mostly in Latvia, Poland and Portugal [18, 26, 27]. The prevalence is higher than 50% in the population. Studies conducted in Northern Europe, apart from Ireland, displayed quite decreased level of *H. pylori* infection [28].

Among the Asia–Pacific region, the distribution of *H. pylori* infection is various between countries, as well as chosen regions within individual communities. This prevalence depends on socioeconomic lifestyle levels, which is lower in more developed regions. In Vietnam, the *H. pylori* seroprevalence rate was 74.6% [29]. In India, the reported rate was even higher, reaching around 79% [30]. Bangladesh was one of the region, highly affected *by H. pylori* occurrence, with 92% of prevalence among population [31]. In Australia, which is more developed region the seroprevalence rate reached 15, 4% [32]. Within East Asian regions, the overall seroprevalence rate was 39.3% in Japan, 54.5% in Taiwan, 58.07% in China and 59.6% in South Korea [33–36]. Southeast Asia countries displayed the infection rate, assigned to 31% in Singapore, 35.9% in Malaysia and 57% in Thailand [37–39].

### Action of *H. pylori* in the stomach

*H. pylori* is the most common infection in the world and the etiological factor causes GCs [40]. In total*, H. pylori* infection almost doubles the overall risk of gastric cancer. GC develops through a cascade of well-defined and well-recognizable steps: inflammation, atrophy, intestinal metaplasia, dysplasia, and carcinogenesis, and is closely associated with the environment, diet and gene mutations [41–44]. The recognition patterns of gastric cancer include genetic alterations among cell cycle regulators, factors that regulates apoptosis, microsatellite instability, multidrug resistance proteins, factors that influence cell membrane properties, module of HER2 expression, and agents with impact on the progression of gastric cancer and peritoneal metastasis [45].

Cells of gastric mucosa produce hydrochloric acid and the digestion of food in the stomach takes place at low pH conditions, but they are not fully acid-resistant. Therefore, under normal conditions, they are covered with a thin layer of protective mucus [46]. Nonsteroidal anti-inflammatory drugs, e.g. acetylsalicylic acid, damage the gastric mucosa and lead to the development of ulcers, among others because they inhibit the synthesis of prostaglandins, which in turn activate the production of mucus in the stomach [47]. *H. pylori* can adapt to the local conditions prevailing in the stomach, especially in the pylorus. It avoids areas with extremely low pH, thus the light of the stomach away from its walls. It migrates instead towards the epithelium of the mucous membrane where it penetrates the mucus layer covering the cell. Among people secreting larger amount of hydrochloric acid, the *bacterium* is more likely to colonize the pylorus, especially in the place where the stomach passes into the duodenum. In this way, the central part of the stomach is avoided, where the pH is clearly low. When hydrochloric acid secretion is lower, *H. pylori* colonizes the whole stomach [48].

Movement of the *bacteria* is active, it occurs due to the characteristic shape. Then *H. pylori* adheres to epithelial cells and even penetrates them. An important factor determining the effects of *H. pylori* infection is the way in which the immune system reacts to the *bacteria*. Another known toxic product of *H. pylori* metabolism is ammonia. It is a weak alkali which helps *bacteria* to neutralize hydrochloric acid in its immediate environment and is the cause of survival. It is made of urea which is produced by urease, the enzyme which *H. pylori* releases [49].

### The geographically dependent pathogenesis of *H. pylori* associated GCs

The genomes of *H. pylori* are incredibly diverse, various virulence factors have impact on host epithelium in different ways, including direct and indirect investigations like eliciting immune response. Genetic alterations of host, smoking, different food consumption, gastric microbiota and long-term intake of proton pump inhibitors (PPIs) impact on the progression of *H. pylori* associated gastric cancers [50]. The pathogenesis of *H. pylori* related gastric lesions is a multi-step and complex action, which the progression is based on combining of environmental, host and bacterial agents [51].

Mechanism of *H. pylori* associated gastric carcinogenesis has not been completely described. *H. pylori* infection commonly lasts for decades, causing a bunch of histological variations, encompassing apoptosis and proliferation of epithelial cells, destruction of intercellular junctions, and malignant transformation [52]. *H. pylori* genetic alterations, outcomes in different *H. pylori* products on gastric epithelium and cellular signalling processes, which have been broadly studied in recent decades [53]. It is assumed that the *bacteria* themselves do not cause mutations that lead to cancer, rather the infection stimulates a local inflammatory reaction, in which there is a white cell infiltration and local production of free radicals, damaging DNA and initiating the development of cancer cells [54]. Another mechanism of developing cancer cells, could result from the death of epithelial cells, damaged by *H. pylori*. The defects are filled by replacing these cells with new, mutated, potentially cancerous cells, which can enter the subdivision cycle [55]. According to Guven-Maiorov et al., 2017 others survey pathogens often interact with the host through proteins. *H. pylori* proteins interfere with multiple host pathways, as they target several host proteins and thereby alter the host signalling. It may mimic host proteins at different stages - the sequence, structure, motif or interface levels [56].

*H. pylori* is a heterogeneous *bacterium* and its virulence factors are different among geographic regions. Various geographic incidence in gastric cancer development might be explained, mainly by the occurrence of different *H. pylori* virulence agents, like CagA, VacA and OipA [57]. *H. pylori* infection also might take part in development of duodenal ulcer. This variance is possibly connected with *H. pylori* virulence factor DupA [58].

CagA is the most popular and broadly studied *H. pylori* virulence factor. There are two types of clinical *H. pylori* isolate: CagA-producing (cagA positive) strains and CagA-nonproducing (cagA negative) strains. In Western countries, it has been postulated that cases infected with cagA-positive strains of *H. pylori* are displaying an increased risk of gastric cancer or peptic ulcer in comparison to those affected by cagA-negative strains [59, 60]. The pathogenic difference in East Asia is not so obvious to explain, by taking into consideration the occurrence or absence of the *cagA* gene alone, as most strains of *H. pylori* have the *cagA* gene irrespective of the disease [61]. It is worth to mention that *cagA* is a polymorphic gene, therefore the various numbers of repeat sequences located in the 3′ region of the *cagA* gene of different *H. pylori* strains are present [62]. Each repeat region of the CagA protein encompasses Glu-Pro-Ile-Tyr-Ala (EPIYA) motifs, containing a tyrosine phosphorylation site. Additionally, each CagA is allotted a sequence type containing the names of the EPIYA segments in its sequence (that is, ABC, ABCC or ABCCC for Western-type CagA and ABD for East-Asian-type CagA) [62, 63, 64].

VacA is the second very popular *H. pylori* virulence factor. Additionally, to promote vacuolation, VacA can start multiple cellular activities, like cytochrome c release from mitochondria causing the apoptosis process, membrane-channel formation, as well as binding to cell-membrane receptors followed by initiation of a proinflammatory response [65, 66]. The difference in the vacuolating actions of different *H. pylori* strains are displayed mostly because of differences in the *vacA* gene structure at the signal (s) region (s1 and s2) and the middle (m) region (m1 and m2) [67]. In Western countries, mostly Latin America, the Middle East and Africa, the studies performed claim that individuals infected with m1 or s1 *H. pylori* strains have a chance for higher risk of developing gastric cancer or peptic ulcer in comparison to those underwent the infection with m2 or s2 strains [68, 69]. In East Asia, *H. pylori* strains have an s1-type s region, that’s why the pathogenesis cannot be examined by the type of s region present [61]. However, m1 strains are mostly present in parts of north East Asia, like Japan or South Korea, m2 strains are popular in parts of south East Asia, such as Taiwan and Vietnam [70, 71].

OipA was firstly described as a proinflammatory-response-inducing protein, mostly because of the role of *oipA* isogenic mutants in reducing the production of IL-8 from gastric epithelial cell lines [72]. *In vivo* performed investigations using human gastric biopsies also results in the same conclusion that a functional oipA was importantly correlated with high mucosal IL-8 levels [60]. Finally, it stayed obvious that one of the OipA role is to promote inflammation and actin dynamics through phosphorylation process of multiple signalling pathways [73, 74]. Additionally, as and adhesin, OipA is committed in the attachment of *H. pylori* to gastric epithelial cells *in vitro* [73]. It is significant to mention that to all East Asian strains are producing functional OipA protein [72].

### *H. pylori* virulence factors and further consequences in gastric carcinogenesis

Cytotoxin associated factor (CagA) and vacuolizing cytotoxin (VacA) are specific strains of *H. pylori*, which are mainly virulence factors involved in increasing the risk for gastric carcinoma development [75]. CagA - protein is a 120- to 140-kDa protein that is translocated into host cells by the type IV cag secretion system, after the attachment of *H. pylori* and in the result changes the cell-signalling mechanisms in gastric cells. VacA protein is a cytotoxin produced by *bacteria* and inducing vacuolation of the epithelial cells. The gene is present in all strains but has different variations of vacuolating activity [76]. This aspect is characteristic to variations in *vacA* gene structures within the three regions: the signal sequence region (s-region) (s1 or s2), mid-region (m-region) (m1 or m2) and the intermediate-region (i-region) (i1, i2 or i3) [40, 77, 78]. These two virulent factors and their polymorphism have been considered in many research [79–82].

In the study Matos et al., 2013 summarized a possible association between these genotypes and the risk for developing different gastric phenotypes. Scientists checked forty-four studies, all with either a case–control (n=13) or cross-sectional (n=31) design, including a total of 17 374 patients: 107 in the dysplasia group, 4994 in the peptic ulcer disease group, 5564 in the gastritis group and 6709 in the gastric cancer group. They confirmed an increased risk for gastric cancer among patients infected with CagA+ *H. pylori* strains and infected with VacA s1 and m1 strains [75]. Pandey et al., 2014 reviewed the comparative study of *H. pylori* infection and the possible influence of carcinogenic CagA+ strains in the progression of gastric cancer. But not all strains of *H.pylori* are pathogenic. The virulence is primarily determined by a gene *cagA* and these strains have strong association with gastric cancer [78, 83, 84]. *H. pylori* virulence factors are listed in Table 1. They discussed the connection between gastric cancer and stimulant substances like tobacco and alcohol. According to their study the number of *H. pylori* infection achieved higher amount in precancerous and cancerous gastric lesions and was related to the habit of tobacco use. It was because the infection was acquired orally [83].

**Table 1:** *Helicobacter pylori* virulence factors and further consequences to GC development

	Risk Factors Action	Consequences	Authors
*H. pylori virulence factors*	Activation and secretion of cytokines in epithelial cells such as IL-8 by *cag* pathogenicity island (*cag*PAI)	• CagPAI*-* and inflammation-driven cancerogenesis • Codetermination of the risk for gastric cancer	Stein et al., 2017 [86]
	Influence of CagA on the tumor suppressor function of apoptosis-stimulating protein of p53 (ASPP2)	• The interaction between CagA and ASPP2 • The consequent degradation of p53 • Increased risk of gastric cancer	Buti et al., 2011 [87]
	CagA-dependent loss of polarity and activation of aberrant *Erk* signalling after the delivery into epithelial cells	• Senescence and mitogenesis in epithelial cells, both nonpolarized and polarized	Saito et al., 2010 [92]
	East Asian-type CagA has a higher binding affinity for the Src homology-2 domain-containing phosphatase 2 (SHP2)	• Greater risk of peptic ulcer development and/or gastric cancer when compared to its Western counterpart	Hatakeyama et al., 2004; Higashi et al., 2002; Jones et al., 2009; Vilaichone et al., 2004 [95–98]
	CagA-positive strains with EPIYA motifs; Strains possessing cagA with an EPIYA-D segment (an East Asian-type cagA-positive strain)	• Reduction variety of intracellular signalling systems after the infection of gastric epithelial cells; • Higher risk of gastric cancer among infected individuals	Yamaoka et al., 2010; Backert et al., 2001 [100, 101]

*H. pylori* which is virulent, can cause proinflammatory signalling which induces the activation and secretion of cytokines in epithelial cells such as IL-8. Cag pathogenicity island is responsible for this signalling. It is the codetermination of the risk for gastric cancer [85]. On the encoded cagPAI the Cag type IV secretion system (CagT4SS), can change the place of various molecules in cells, the effector protein CagA, metabolites and DNA. Although these translocated molecules are known to contribute to cellular responses to some extent, a major part of the cagPAI-induced signalling leading to IL-8 secretion remains unexplained [86]. Stein et al., 2017 reported that biosynthesis of an intermediate metabolite of LPS inner heptose core, heptose-1,7-bisphosphate (HBP), is translocated into host cells dependent on the CagT4SS. It is also the main factor which leads to the activation of cellular responses and contributes in the induction of proinflammatory signal and IL-8 secretion in human epithelial cells. This response pathway is linked with the human cellular adaptor protein TIFA. The knowledge of this will enable to understand the way of modulating the immune response in human host by *H. pylori*. Mutants defective in the genes required for synthesis of HBP exhibited a more than 95% reduction of IL-8 induction and impaired CagT4SS-dependent cellular signalling. It was not possible to enrich the active metabolite in bacterial supernatants, but it was present in *H. pylori* lysates. These results will help to understand *H. pylori* cagPAI-dependent signalling which is mediated by intracellular pattern recognition receptors. Stein et al., 2017 also advance the knowledge of immunomodulatory activities by *H. pylori* and make the possibilities of intervention in cagPAI- and inflammation-driven cancerogenesis more realistic [86]. The Cag Type IV secretion system, which leads to inflammation and cancerogenesis during chronic infection, is one of the main virulence factors of the bacterial gastric pathogen *H. pylori*. However, the mechanisms lead to cagPAI-dependent signal transduction and cytokine.

Buti et al., 2011 disproved that cytotoxin-associated gene A has influence on the tumor suppressor function of apoptosis-stimulating protein of p53 (ASPP2) [87]. Different pathogens which may cause persistent infections have the property to demonstrate strategies which can interfere with signalling pathways and immune responses of the host. Type I strains of *H. pylori* encode a type four secretion system that allows the pathogen to produce the effector protein (CagA) straight to the host cells [88, 89, 90, 91]. It is the most important factor which connects the infection of the *bacteria* with the gastric cancer. After translocation into the host cell, CagA increases cell motility, changes cells shape, and promotes transition-like phenotype from an epithelial to mesenchymal. CagA leads to the damage of p53 and its activity in an ASPP2-dependent manner is down-regulated. Cells which are infested with *H. pylori* treated with drug Doxorubicin with activated the p53 are more resistant to apoptosis than uninfected cells. They require this effect from ASPP2. This is an example of bacterial protein that disproves the *p53* tumor suppressor pathway in a manner like DNA tumor viruses [87].

The findings made by Saito et al., 2010 suggest that CagA uses a polarity-signalling pathway to cause oncogenesis. CagA produced by *H. pylori* constitutes a very important factor in gastric carcinogenesis. It induces loss of polarity and activates aberrant *Erk* signalling after the delivery into epithelial cells. Scientists claimed that CagA-induced *Erk* activation effects on the senescence and mitogenesis in epithelial cells, both nonpolarized and polarized [92].

In the study Subsomwong et al., 2017 compared the amount of incidence in gastric cancer in Thailand regions, in the North and South [93]. They showed that the prevalence of *H. pylori* infection was three times higher in patients living in the North (50.4%) than in the South (17.6%) [93, 94]. However, they claimed that those in the South had more severe lesions of precancerous and cancerous stages of gastric cancer. To check the virulence genotypes of *H. pylori* they made further analysis. The outcome which they achieved was that patients from the South may have a higher amount of risk to induce gastric cancer because of the infection with *H. pylori*. In the South there are Western-type CagA strains which could develop more precancerous lesions and histopathological changes in the antrum and the corpus of the gaster even after checking the differences between age, sex, location, and birthplace. The second repeat of the C-terminal region of CagA sequences has a difference between East Asian-type CagA and Western-type CagA. East Asian-type CagA has a higher binding affinity for the Src homology-2 domain-containing phosphatase 2 (SHP2), resulting in a greater risk of peptic ulcer development and/or gastric cancer when compared to its Western counterpart [95–98]. The scientists checked the ability of gastric cells to induce IL-8. They did not find the relationship between the *cagA* genotypes and the location on IL-8 production. However, they agreed that further studies must be made to measure other pathological markers than IL-8.

Aftab et al., 2017 focused on Bangladesh population with high number of *H. pylori* infection but a low morbidity to gastric cancer. *H. pylori* strains can be divided in two groups cagA-positive or -negative [99]. CagA expression in cagA-positive strains relates to inflammation and the higher risk for more severe clinical outcomes against to cagA-negative strains [100]. CagA-positive strains that have EPIYA motifs, tyrosine-phosphorylated by Src and Abl family kinases, reduce a variety of intracellular signalling systems after the infection of gastric epithelial cells [101]. There is a higher risk of gastric cancer among individuals infected with strains possessing cagA with an EPIYA-D segment (an East Asian-type cagA-positive strain) than with strains possessing an EPIYA-C segment (a Western-type CagA-positive strain) [100]. The scientists analysed CagA and VacA subtypes and their association with severe histology type. They segregated Bangladeshi strains into two populations of different genotypes. They claimed that in *H. pylori* populations, hpAsia2 strains were associated with higher activity and inflammation in the antrum than in the hpEurope strains. The cause of lower level of gastric cancer among Bangladesh population might be the high number of less-virulent genotypes. It could be a better risk factor of gastric cancer than the ancestral origin of the *H. pylori* strains. Moreover, the *vacA* m region may be use as a better virulence marker than other regions [99].

### Genetic variants and *H. pylori* associated gastric cancer

Genetic variants in genes from cytokines and their receptors associated with inflammation are perceived to take part in tumor initiation and promotion. Considering genetic polymorphisms in gastric cancer and *H.pylori* infection, the growing interest in this field has become more expanded in recent years. In this process different cytokines take part e.g. *IL-1, IL-17* (it is associated with a higher risk of developing gastric cancer connected with *H. pylori* colonization), tumor necrosis factor (*TNF*) α, toll-like receptors (*TLRs*) [77, 102–114]. The next occurrence of SNP’s is protection against pathogens which attacks (*MUC1*), the repair of DNA damage caused by *H. pylori* (*XPA, XPC, ERCC2*) and cell-to-cell adhesion (CDH1) [13, 94, 115–121]. SNP’s also has influence on metabolic processes of foliate (methylenetetrahydrofolate reductase), polycyclic aromatic hydrocarbons (*GSTT1, SULT1A1, NAT2, EPHX1*), hormones e.g. estrogen and androgen and xenobiotics (*Cyp2e1*) [122–126].

At a gene level, *hypoxia-inducible factor 1* (*HIF-1*) is the primary transcriptional activator, very sensitive to oxygen, helping cells to survive in low oxygen tension [127]. Overexpression of *HIF-1α* is important in the activation the bunch of genes involved in cancer biology, encompassing cell proliferation, survival and apoptosis, glucose metabolism, erythropoiesis, as well as angiogenesis [128]. It was displayed that the expression of around 20 genes is regulated by *HIF-1α*, including *NFκB1*, which is involved in regulation of inflammation and cancer [129].

It was postulated that the transmembrane protein CD133 is overexpressed in 57% of gastric cancer and positively connected with the expression of Ki-67 [130]. One of the member of the cadherin family, CDH17, was described to be a marker for gastric cancer in early stage [131]. The overexpression of CD168 was assigned to be correlated with the depth of cancer invasion and metastasis stage [132]. In another investigation, Xie et al., 2015 described that tumor stem cell surface marker CD44 (CD44v6), which relates to metastasis in GC [133].

Matrix metalloproteinases (MMPs) are enzymes involved in multiple processes, covering degradation of extracellular matrix, inflammation, tumor invasion and metastasis. The meta-analysis performed by Long et al., 2014 displayed significant increment of MMP-7 expression in GC cases, also the positive correlation with lymph node metastasis and invasion of the tumor [134].

Epithelial–mesenchymal transition (EMT) it is important in tumor progression and invasion and *Snail* regulates the EMT in different carcinomas. The *Snail* overexpression was observed to be correlated with increased invasion, cell migration and tumor progression in gastric cancer [135].

Other cytokines like tumor necrosis factor α (*TNF-α*), *IL-6, IL-8, IL-10*, Toll-like receptor 4 (*TLR-4* and transforming growth factor β (*TGF-β*) are suspected to affect tendency to be infected by *H. pylori* or to change the pathways of local inflammation [86, 136–138]. However, it is possible that several low-penetrant alleles in combination may result in familial aggregation, rather than dominant cancer genes with high penetration [139].

**Figure 1:**
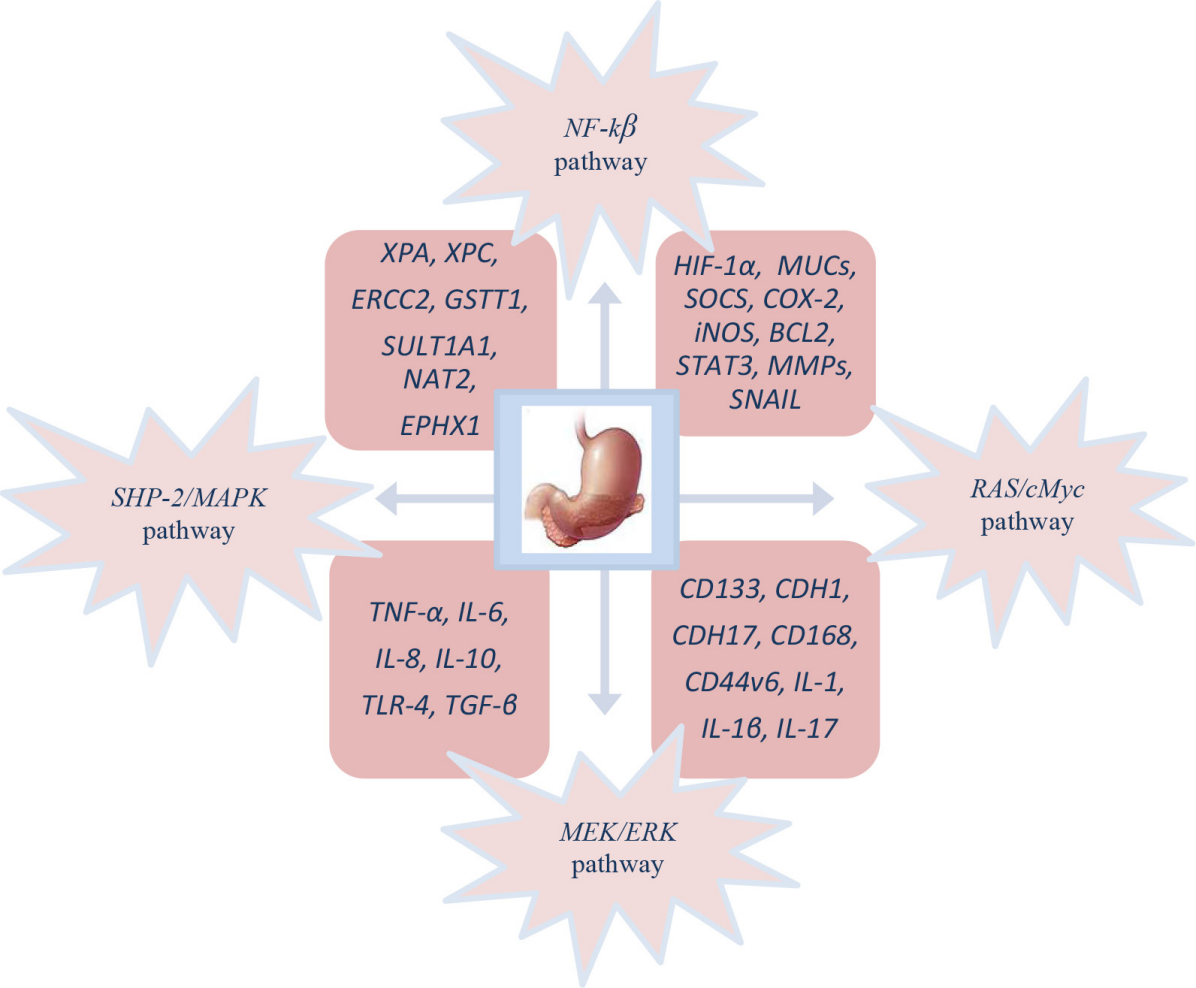
Molecular pathogenesis of GC patient’s infected with *H. pylori*

Cellular and molecular pathogenesis of *Helicobacter pylori* infection in gastric carcinogenesis is shown on Figure 1. The phosphorylated *cagA* active in the *SHP-2/MAPK* pathway regulates *NF-κB, RAS*/*cMyc* and *MEK*/extracellular signal-regulated kinase (*ERK*) pathways, which is the result of the regulation of genes like *HIF-1α, MUCs*, inducible nitric oxide synthase (*iNOS*), *BCL2*, suppressor of cytokine signalling (*SOCS*), signal transducer and activator of transcription 3 (*STAT3*), *COX-2*, *MMPs* and *SNAIL* provoking proliferation, differentiation, increased migration, invasion and metastasis of cancer cells [140].

Epidemiological studies indicated the connection between the *H. pylori* infections and the gastric mucosa inflammation, which change into chronic gastritis and intestinal type of gastric cancer. *Interleukin-1β (IL-1β)* deregulation is responsible for the higher risk of gastric carcinoma (Table 2) [40]. The latest studies claimed that the *IL-1β-31* and *-511T* alleles links to hypochlorhydria and precancerous lesions, so they are closely associated with carcinogenesis process, but the leading mechanism is still unknown. *H. pylori* infection generates IL-1β and suppresses the secretion acid in the gastric mucosa while the eradication decreases the expression of IL-1β [141, 142]. El-Omar et al., 2003 first reported an association between the *IL-1β-31C* and *IL-1RN 2/2* genotypes with a greater risk of chronic low gastric acid secretion and gastric cancer [143]. The presence of IL-1β-511T, IL-1RN*2/*2, TNF-α-308A, and *IL-10* (haplotype *ATA/ATA*) was connected to a higher risk of noncardia gastric cancer with *H. pylori* infection [86, 138]. Moreover, Hong et al., 2016 found that *H. pylori* infection has a similar effect on the evolution of gastric cancer with *IL-1β* gene polymorphisms, and the highest amount of severe gastric anomalies occur in patients with high-risk genotypes (*cagA(+)/vacAs1(+)/IL-1β-511T*) both host and bacterial [40]. *H. pylori* synergistic with *IL-1β* gene polymorphisms seem to promote gastric cancer by their involvement in precancerous gastric lesions and hypochlorhydria. The histological type of gastric cancer may be also dependent on *IL-1β* [144, 145]. Intestinal type against to the diffuse or mixed-type of gastric cancer is more often in those with *IL-1β-511T* genotype [144, 146–148]. What is more, the levels of mucosal *IL-1β* are higher in *H. pylori-*infected gastric cancer patients with *IL-1β-31TT* as against those with *IL-1β-31CT* and *IL-1β-31CC* [149]. However, the infection by *H. pylori* is more likely among patients with *IL-1β-31 CT* and *TT* genotypes in regions of Asia and Latin America than in those with *IL-1β-31 CC* [121]. A higher number of intestinal metaplasia occurs in patients with *H. pylori* infection, especially in those with *vacA* m1 strain [150]. *H. pylori* induces the expression of *IL-1β*, which starts gastric carcinogenesis by affecting two types of cells inflammatory and epithelial [151]. But scientists agreed that further research is needed to expand the study area in different populations and subtypes of gastric cancer and to explain alternative underlying mechanisms.

**Table 2:** *IL-1β* genotypes and higher risk of gastric carcinoma

Genotypes	Phenotypes	Authors
*IL-1β-31* and *-511T*	Induction of hypochlorhydria and precancerous lesions	[Takashima et al., 2001; Wang et al., 1999] [141, 142]
*IL-1β-31C* and *IL-1RN 2/2*	Greater risk of chronic low gastric acid secretion and gastric cancer	[El-Omar et al., 2003] [143]
IL-1β-511T, IL-1RN*2/*2, TNF-α-308A, and *IL-10* (haplotype *ATA/ATA*)	Higher risk of noncardia gastric cancer	[Stein et al., 2017; Sokolova et al., 2014] [86, 138]
(*cagA(+)/vacAs1(+)/IL-1β-511T*) both host and bacterial	Severe gastric anomalies	[Hong et al., 2016] [40]
*IL-1β-511T*	Frequent occurrence of intestinal type against to the diffuse or mixed-type of gastric	[Yu et al., 2010; Kamangar et al., 2006; Ruzzo et al., 2005; Wang et al., 2007] [144–148]
*IL-1β-31TT*	Higher levels of mucosal IL-1β in *H. pylori-*infected gastric cancer patients	[Chang et al., 2005] [149]
*IL-1β-31 CT* and *TT*	Increased possibility of infection by *H. pylori*	[Sun et al., 2015] [121]

### *H. pylori* and early-onset gastric cancer

**Figure 2:**
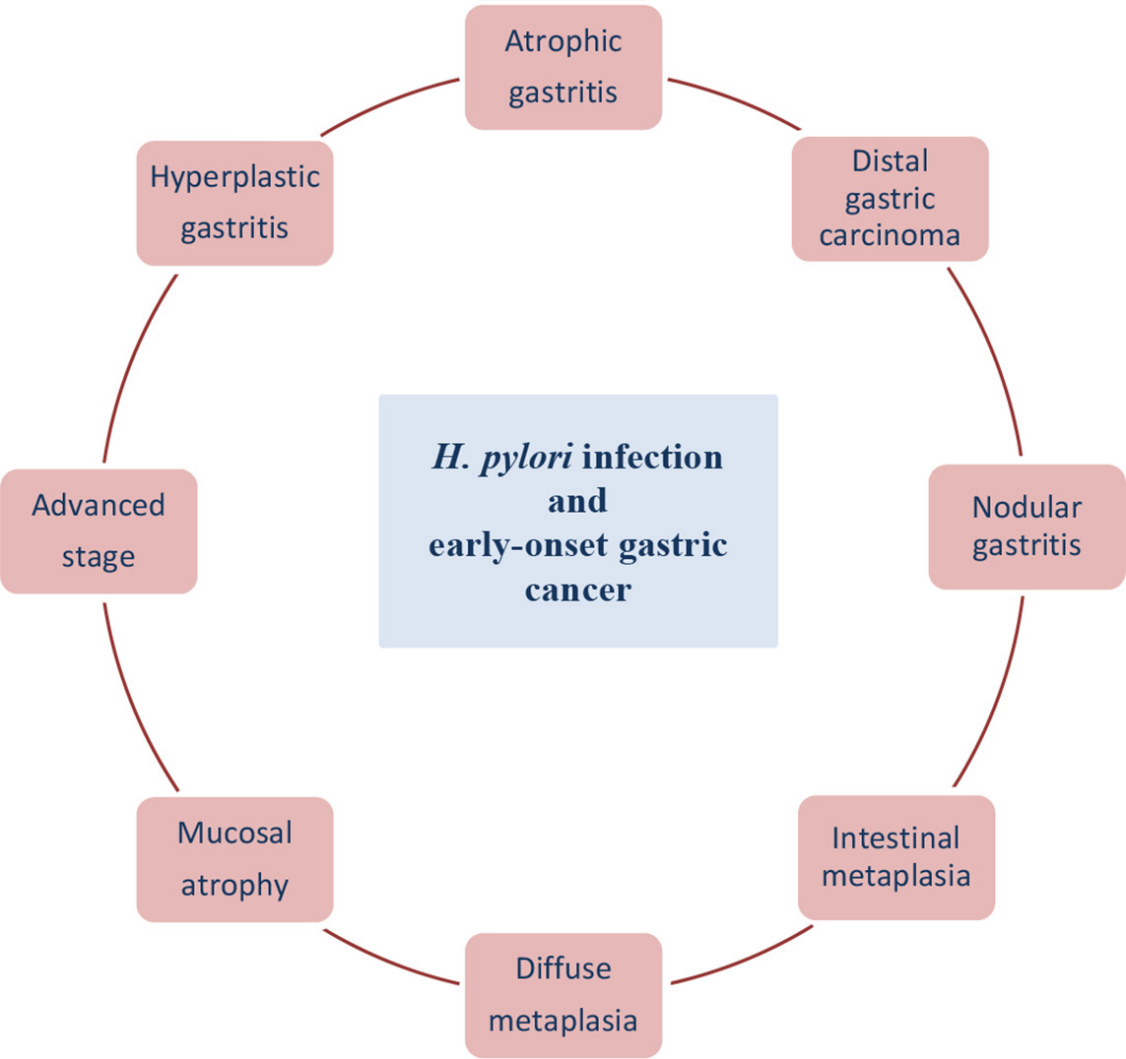
*H. pylori* infection at the age before 45 years

It is considered that gastric cancer arises as a combination of environmental and genes factors and affects mainly older patients. An inherited component contributes to <3% of gastric cancers and most of genetic changes associated with gastric cancer are acquired [120]. Over the past few decades, advances in technology and high-throughput analysis have improved understanding of the molecular aspects of the pathogenesis of gastric cancer but the categorization of carcinogenic events is highly complicated. The current scientific challenge is to distinguish which alterations of GC are crucial, what is the connection between these alterations and how to prevent their incidence [120, 152]. Gastric cancer at the age before 45 years (without cancer history in the family) is very rare and is called early-onset gastric carcinoma (EOGC). It is thought that these patients develop gastric cancer with a molecular genetic profile which is different from that occurring at a later age. What is more the impact of environmental factors being less important [6]. *H. pylori* positive gastritis is considered to occur among different gastric cancer forms in young patients, like nodular gastritis, atrophic and hyperplastic gastritis, diffuse and intestinal metaplasia, mucosal atrophy, distal GC and advanced stage [153–156] (Figure 2). This chapter describes the particular role of *H. pylori* infection and early-onset gastric cancer development.

Host-related factors have been more relevant than impact of environment factors in developing early-onset gastric cancers. In the study conducted by Rugge et al. 1999 [156], the possible correlation between *H. pylori* infected patients by cagA positive strains was shown as an important factor in developing gastric carcinoma among young patients. The group of 105 patients with gastric cancer in the age between 16-40 years, as well as control group were analysed. The *H. pylori* infection occurrence was assessed by histological analysis as well as using standard PCR method to confirm *cagA* genotype and *ureC* gene, which are indicators of *H. pylori* infection. The important correlation between intestinal, as well as diffuse GCs and infection by *H. pylori* was confirmed among young Italian, where the *bacterium* has an etiologic significance in both types of GC [156].

The *H. pylori* infection is higher in older patients in the asymptomatic population in Japan and is significantly increased in GC cases older than 40 years [154]. The researchers were interested in finding the association between *H. pylori* positive patients younger than 40 years. The group of 40 Japanese GCs in age below 40 years were compared with the equal number of controls. *H. pylori* infection in gastric mucosa was observed to be importantly increased among patients with cancer in comparison to control group. Moreover, the analysis was performed with 18 cases of intestinal type of young GCc and 70 cases of diffuse, showing the connection between acute, chronic inflammation and mucosal atrophy, intestinal metaplasia in the background of mucosa in both analysed types of GC, with the higher level when compared to the control group.

According to Marcos-Pinto et al., 2013 first-degree relatives of early-onset gastric cancer is supposed to be a population with a different molecular and phenotypic profile, in connection with the precancerous stages of gastric cancer. *H. pylori* was present in 82% of cases (vs 62% in controls; p=0.004) with *vacA* s1 and *vacA* m1 + strains with the presence of atrophy. They showed the increased prevalence of high-risk of precancerous conditions, which may be associated with high virulence *H. pylori* strains and pro-inflammatory host genotypes [157].

Masuda et al., 2008 [155] found that gastric cancer occurring in young age is significantly correlated with the *H. pylori* infection but not with the family history or the genetic background. The patients with gastric carcinoma had an increased level of *H. pylori* infection than those with normal mucosa. Additionally, nodular gastritis, atrophic gastritis and hyperplastic gastritis were assigned using endoscopy method as closely correlated with the *H. pylori* positive gastritis. Polymorphism of *P4502E1 (CYP2E1)* and family history were not variable among different age groups.

*Helicobacter pylori* infection in young population of patients with diagnosed GC was investigated by Kokkola et al., 1996 [158]. Biopsy samples were analysed among 50 cases of GCs, up to 45 years old, compared to controls, and *H. pylori* infection was confirmed by immunostaining. Obtained results showed that *bacterium* is a risk factor of early-onset gastric cancer, was found in 72% of GC cases and 43% of controls. The association between infection with CagA + and CagA - strains of *H. pylori* and young patients with GCs was investigated by Kikuchi et al., 1999 [153]. Among Japanese population, CagA IgG antibodies were measured in sera of 101 GC patients younger than age 43, 103 <40 years old and 100 in patients with benign diseases. In comparison to the *H. pylori* - /CagA - cases, both the *H. pylori* + /CagA - and the *H. pylori* + /CagA + groups revealed high odds ratios for early, advanced, distal, intestinal and diffuse type of GC.

## CONCLUSIONS

*Helicobacter pylori* is a gastric pathogen that colonizes around 50% of the world's population. *H. pylori infection* provokes mostly chronic inflammation, as well as importantly increases the chance of developing duodenal and gastric ulcer disease and gastric cancer. Infection with the *bacterium* is one of the risk factor for GC, which is the fourth leading cause of cancer-related deaths worldwide. It is now clear that cancer risk is the combining effect of the polymorphic nature of the host genotype, bacterial population in the host, as well as environmental factors. Based on the current knowledge, encompassing genome sequences, both human and *H. pylori*, detectable phenotypes (CagA phosphorylation) and animal models, might be possible to describe fundamental biological basis of *H. pylori*-associated cancer, especially in early age, which should have direct clinical applications. Currently it is important to expand the knowledge about the pathogenesis of *H. pylori-*provoked gastric adenocarcinomas, not only to apply more precise treatments, but also because it might deliver the paradigm for the impact of chronic inflammation on the genesis of other malignancies that developed within the gastrointestinal tract.
